# Cost per Response of Acthar® Gel vs Standard of Care for the Treatment of Proteinuria in Nephrotic Syndrome Due to Idiopathic Membranous Nephropathy Among Adults from the US Healthcare Perspective

**DOI:** 10.36469/001c.142078

**Published:** 2025-08-06

**Authors:** Jas Bindra, Ishveen Chopra, Kyle Hayes, John Niewoehner, Mary P. Panaccio, George J. Wan

**Affiliations:** 1 Falcon Research Group, North Potomac, Maryland; 2 Manticore Consultancy, Bethesda, Maryland; 3 Mallinckrodt Pharmaceuticals, Bridgewater, New Jersey

**Keywords:** Acthar® Gel, cost-per-response, idiopathic membranous nephropathy, nephrotic syndrome, proteinuria, repository corticotropin injection

## Abstract

**Background:** Proteinuria, a critical marker of glomerulosclerosis, poses a challenge in idiopathic membranous nephropathy (iMN), particularly when standard treatments fail. Acthar® Gel, a US Food and Drug Administration–approved treatment option, may offer an alternative for managing refractory proteinuria in nephrotic syndrome (NS) due to iMN where multiple treatments have failed. **Objective:** The cost per response of Acthar® Gel vs standard of care (SoC; cyclophosphamide or rituximab) for treatment of proteinuria in NS due to iMN was evaluated among adults who had failed multiple treatments from a US payer perspective over a 1- to 3-year horizon. **Methods:** A probabilistic, cohort-level state-transition model simulated patient progression through various health states using 6-month cycles. Patients began in a relapse phase and received either Acthar® Gel or SoC. Transition probabilities determined whether patients achieved a response, experienced no response, progressed to renal failure, or remained in relapse. Responders could potentially maintain their response or relapse, while nonresponders risked renal failure, with potential mortality from any state. Clinical, healthcare resource utilization, and cost data were derived from published literature. Drug prices were based on wholesale acquisition costs. **Results:** Over 1 year, Acthar® Gel showed a lower cost per response (377 185)thancyclophosphamide(551 687) and rituximab ($741 373). This cost advantage of Acthar® Gel was maintained over 2 and 3 years. Acthar® Gel had higher drug acquisition costs than cyclophosphamide and rituximab but resulted in lower overall medical costs and higher response rates within 1 year, without additional treatment-related costs. Over 2 and 3 years, Acthar® Gel had a lower overall cost of care and higher response rates than SoC, establishing it as a dominant treatment option. **Conclusions:** Based on current model assumptions and clinical inputs, Acthar® Gel may potentially be a cost-effective and value-based treatment strategy vs unapproved SoCs for adults with refractory proteinuria in NS due to iMN, particularly for those who have not responded to conventional therapies over a 1- to 3-year period within a US payer context. These results may inform clinical and payer decision-making in cases when other standard therapies fail to achieve desired outcomes for a specific population.

## INTRODUCTION

Idiopathic membranous nephropathy (iMN), a leading cause of nephrotic syndrome (NS) in adults, accounts for approximately one-third of biopsy-confirmed diagnoses, as evidenced by a longitudinal study in the US spanning 3 decades.^1^ The trajectory of iMN is significantly influenced by the magnitude and duration of this protein excretion.^2^ Long-term renal outcomes, specifically 10-year kidney survival, are typically observed within a range of 60% to 80%.^3^ Moreover, the associated NS can lead to significant health issues, including an increased risk of blood clots, fluid retention, and elevated lipid levels, potentially contributing to substantial patient morbidity and mortality.^4,5^

Clinically, iMN often manifests with substantial protein loss in the urine, referred to as proteinuria, a hallmark of NS.^2,3,6^ Substantial protein loss in the urine is a recognized predictor of progressive kidney dysfunction.^7^ Consequently, achieving remission of proteinuria is a central goal of therapeutic intervention.^3^ Research has shown that patients experiencing complete or partial remission demonstrate a slower decline in renal function and a reduced risk of end-stage renal disease (ESRD).^3^ Notably, complete remission is associated with improved long-term kidney and patient survival.^3^ A partial remission also significantly reduces the risk of ESRD progression; even partial remission provides significant protection against ESRD progression.^3^

iMN is also characterized by a notable recurrence rate, with up to 30% of patients experiencing relapse.^6^ Hence, managing proteinuria in NS due to iMN, particularly in cases refractory to standard treatments, presents a significant clinical challenge.^6–8^ The absence of a universally accepted optimal treatment strategy for proteinuria in NS due to iMN among patients who have failed multiple treatments is attributed to the complex and varied underlying disease mechanisms and lack of robust, controlled clinical trials.^6,8^ Glucocorticoids remain a standard first-line therapy for glomerular diseases presenting with proteinuria; however, their prolonged use can lead to substantial adverse effects, including bone density loss, steroid-induced diabetes, and weight gain.^9,10^ Alternative therapeutic agents, such as calcineurin inhibitors, mycophenolate mofetil, cyclophosphamide, and rituximab, have been explored with varying success.^2,6,8^ However, these agents often carry significant side effects, such as increased susceptibility to infections and potential relapse upon discontinuation, contributing to disease progression.^2,6,8^

Approximately one-third of patients with NS due to iMN experience relapse and continue to exhibit persistent or recurrent proteinuria despite standard immunosuppressive therapy, making them the primary candidates for alternative treatments such as Acthar® Gel. Acthar® Gel (repository corticotropin injection, Mallinckrodt Pharmaceuticals) is approved by the US Food and Drug Administration (FDA) to induce a diuresis or a remission of proteinuria in NS without uremia of the idiopathic type or due to lupus erythematosus.^11^ It is a naturally sourced complex mixture of adrenocorticotropic hormone analogs and other pituitary peptides that interacts with all 5 melanocortin receptors.^11^ This interaction suggests that its therapeutic effects may be mediated through multiple anti-inflammatory pathways, both glucocorticoid-dependent and glucocorticoid-independent.^11^ Clinical studies have reported the effectiveness of Acthar® Gel in reducing or inducing remission of proteinuria in NS due to iMN, with safety outcomes characterized.^2,8,12–14^ With an established side effect profile and a higher likelihood of inducing remission,^2,8,12–14^ Acthar® Gel may help appropriate patients with proteinuria in NS due to iMN.

To make informed decisions, clinicians and payers must evaluate clinical outcomes, healthcare resource use, and associated costs of any treatment. This study assessed the cost per response of Acthar® Gel compared with the standard of care (SoC; cyclophosphamide or rituximab) for treatment of proteinuria in NS due to iMN among adults who have not responded to conventional therapies from a US healthcare payer perspective over a 1- to 3-year horizon.

## METHODS

### Model Structure

A probabilistic, cohort-level state-transition model was developed to assess the economic value of Acthar® Gel in treating proteinuria in NS due to iMN in patients who have not responded to conventional therapies. The model compared the cost per response of Acthar® Gel with that of the SoC, comprising cyclophosphamide and rituximab. Although these SoC agents are recommended in treatment guidelines,^6^ their supporting evidence is limited, and they are utilized off-label. Therefore, their safety and efficacy have not been established by the FDA for this indication. The analysis was conducted from a US healthcare payer perspective over a 1- to 3-year period. This study employed Microsoft Excel 2024 to construct and execute the analytical model.

**Figure 1.**
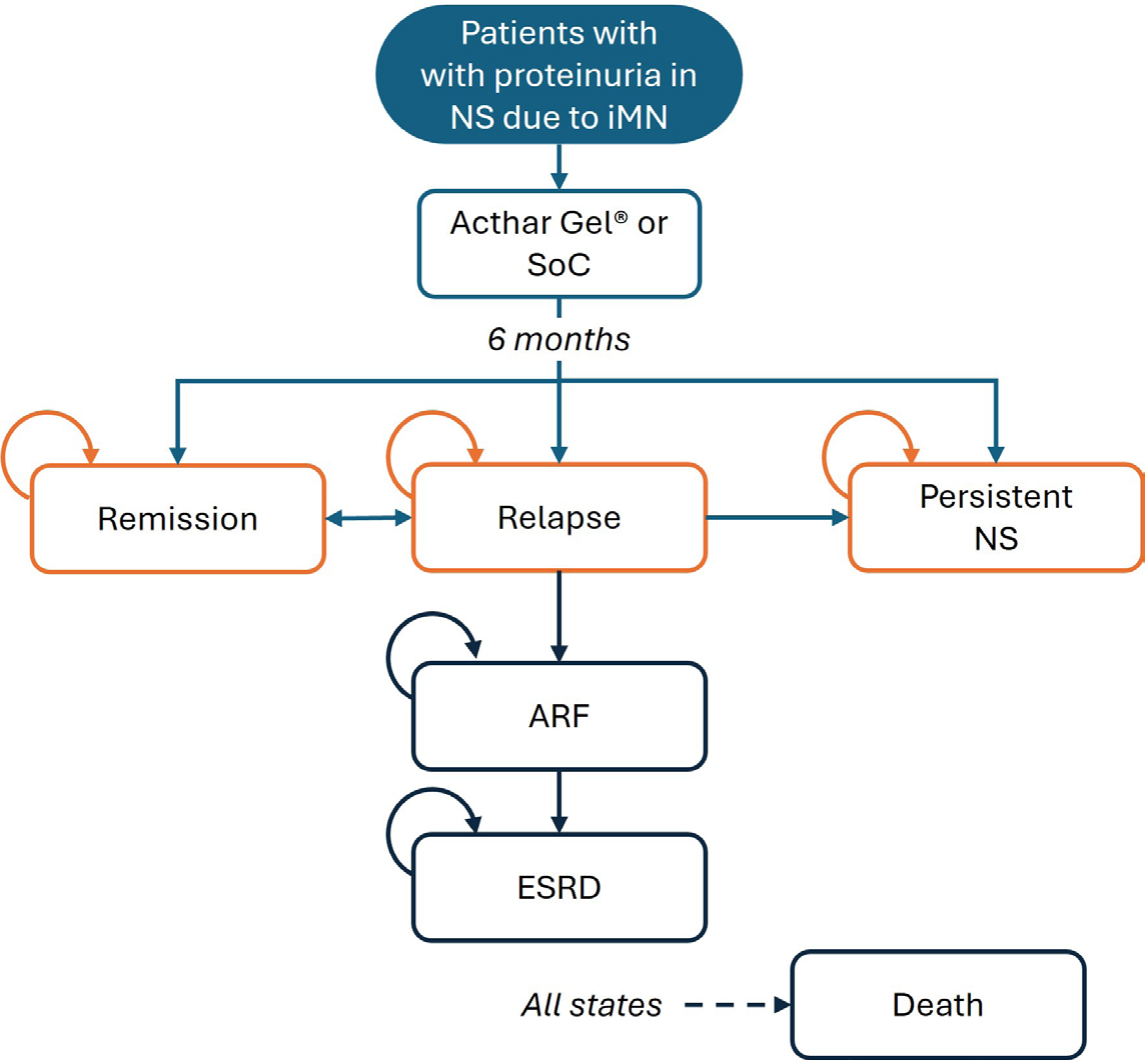
Schematic of the Probabilistic Cohort-Level State-Transition Model for the Treatment of Proteinuria in NS Due to iMN Abbreviations: ARF, acute renal failure; ESRD, end-stage renal disease; iMN, idiopathic membranous nephropathy; NS, nephrotic syndrome; SoC, standard of care. Persistent NS health state refers to the state to which a patient transitions when a patient fails to show a significant clinical improvement following the full course of the prescribed initial treatment.

Patient progression through various health states was simulated, integrating uncertainties in the input data (**Figure 1**). Initially, all patients entered the model in a relapse phase and received either Acthar® Gel or SoC treatment. The probability of treatment success was applied during the 6-month cycles, consistent with current clinical guidelines and best practices, and patient outcomes were evaluated at the end of each cycle. Following treatment in the relapse phase, patients could move to a response state, move to a persistent NS health state, develop acute renal failure (ARF), or have an unresolved relapse. Patients who discontinued treatment or failed to achieve a clinical response with the assigned therapy were assumed to move to a “persistent NS” health state. Patients who continued to relapse (recurrence of proteinuria after treatment) but demonstrated clinical improvement without meeting formal response criteria, were assumed to receive ongoing treatment. Responders could maintain a sustained response or relapse. Patients in the persistent NS health state were at risk of progressing to renal failure, necessitating dialysis or renal transplantation. Death was possible from any health state.

### Model Inputs

**Clinical inputs**: Clinical inputs, including effectiveness, safety, complications, pain, depression, disease progression, and mortality, are presented in **Table 1**.

**Table 1.** Model Inputs: Clinical Inputs and Costs

**Parameters^a^**	**Value ($) or Probability of Event (%)**	**Source**
Effectiveness		
Acthar® Gel		
Response	60.0%	Hladunewich et al^2^^,d^
No response^b^	5.0%	Hladunewich et al^2^^,d^
Persistent NS^c^	35.0%	Hladunewich et al^2^^,d^
Relapse	0.1%	Assumption^e^
Cyclophosphamide		
Response	30.8%	Calculated^f^
No response^b^	36.1%	Calculated^f^
Persistent NS^c^	33.1%	Calculated^f^
Relapse	7.5%	Xue et al^15^
Rituximab		
Response	23.0%	Ruggenenti et al^16^
No response^b^	33.2%	Ruggenenti et al^16^
Persistent NS^c^	43.8%	Ruggenenti et al^16^
Relapse	5.0%	Xue et al^15^
Serious adverse events^g^		
Cyclophosphamide		
Anemia/leukopenia	30.0%	Fernández-Juárez et al^17^
Venous thrombosis	12.0%	Fernández-Juárez et al^17^
Acute kidney injury	19.0%	Fernández-Juárez et al^17^
Infections	23.0%	Fernández-Juárez et al^17^
Rituximab		
Infusion-related adverse events	25.0%	Fervenza et al^18^
Cardiovascular events	1.5%	Fervenza et al^18^
Increased creatinine levels	6.0%	Fervenza et al^18^
Endocrine/metabolic events	3.1%	Fervenza et al^18^
Infections	6.2%	Fervenza et al^18^
Clinical manifestations		
Complications		
Chronic kidney disease	63.0%	Hayes et al^19^
Cardiac complications	64.1%	Rice et al^20^
Pain		
Relapse	58.0%	Lambourg et al^21^
Persistent NS^c^	63.0%	Lambourg et al^21^
Hemodialysis	57.1%	Moore et al^22^
Peritoneal dialysis	58.3%	Moore et al^22^
Renal transplant	46.0%	Lambourg et al^21^
Depression		
Relapse	10.0%	Hayes et al^19^
Persistent NS^c^	21.0%	Shirazian et al^23^
Renal failure	22.8%	Shirazian et al^23^
Disease progression		
Relapse to ARF	5.8%	Troyanov et al^3^
Persistent NS^c^ to ARF	10.7%	Troyanov et al^3^
Persistent NS^c^ to ESRD	8.0%	Kolb et al^24^
ARF to ESRD	4.2%	Coca et al^25^
Mortality		
Remission	1.3%	Kolb et al^24^
Relapse or persistent NS^c^	2.2%	Kolb et al^24^
ARF	8.7%	US Renal Data System^26^
ESRD	7.2%	US Renal Data System^26^
Direct medical costs (annual)^h,i^		
Supportive care medications		
ACEi/ARBs	$149	Micromedex Red Book^27^
Beta-blockers	$304	Micromedex Red Book^27^
Calcium channel blockers	$380	Micromedex Red Book^27^
Diuretics	$97	Micromedex Red Book^27^
Anticoagulants	$629	Micromedex Red Book^27^
Statins	$148	Micromedex Red Book^27^
Immunosuppressants		
Systemic corticosteroids	$4383	Micromedex Red Book^27^
Cyclosporine	$16 810	Micromedex Red Book^27^
Tacrolimus	$16 843	Micromedex Red Book^27^
Mycophenolate mofetil	$1527	Micromedex Red Book^27^
Plasmapheresis	$4888	Micromedex Red Book,^27^ Heatwole et al,^28^ Winters et al,^29^ US BLS^30^
Pain medications		
Pain medication use		
NSAIDs	$2242	Katz et al^31^
Opioids	$4071	Ding et al^32^
Pain medication-related toxicity		
NSAIDs-related toxicity	$916	Rahme et al^33^
Opioid-related substance abuse	$50 453	White et al^34^
Antidepressants use	$3483	Glassman et al^35^
iMN-related disease management		
Inpatient	$9340	Nazareth et al^36^
Outpatient	$17 260	Nazareth et al^36^
Renal failure management		
Hemodialysis	$188 520	Bhatnagar et al^37^
Peritoneal dialysis	$174 188	Bhatnagar et al^37^
Renal transplant	$487 836	Bentley et al^38^
Complications		
Chronic kidney disease	$17 664	Jha et al^39^
Major cardiac complications	$21 683	Jha et al^39^
End-of-life care	$105 124	French et al^40^
Serious adverse events (per event)		
Anemia/leukopenia	$23 872	Weycker et al^41^
Venous thrombosis	$19 711	Grosse et al^42^
Infections	$28 968	Owens et al^43^
Infusion-related	$9386	Foley et al^44^
Cardiovascular	$29 467	Afana et al^45^
Endocrine/metabolic or impaired fasting glucose and diabetes mellitus	$15 519	Fingar et al^46^
Acute kidney injury/increased creatinine level/reversible nephrotoxicity	$49 688	Stottlemyer et al^47^
Irreversible nephrotoxicity	$180 819	Stottlemyer et al^47^
Bleeding	$636	Heatwole et al^28^
Catheter occlusion	$2477	Heatwole et al^28^
Corticosteroid-related toxicity (annual)	$87 592	Rice et al^48^

Abbreviations: ACEi, angiotensin-converting enzyme inhibitors; ARBs, angiotensin receptor blockers; ARF, acute renal failure; BLS, Bureau of Labor Statistics; ESRD, end-stage renal disease; iMN, idiopathic membranous nephropathy; NS, nephrotic syndrome; USD, US dollars.

^a^All parameters from the literature were converted to a 6-month probability, where applicable.

^b^No response refers to patients who initially did not exhibit a therapeutic effect but subsequently achieved a response and experienced resolution of their relapse.

^c^Persistent NS health state refers to the state to which a patient transitions when a patient fails to show a significant clinical improvement following the full course of the prescribed initial treatment.

^d^Complete remission was defined as proteinuria <0.3 g/day and partial remission as a reduction in proteinuria by >50% with a final urine protein <3.5 g/day, but >0.3 g/day and no response as a reduction in proteinuria by <50% or worsening of proteinuria.

^e^A conservative relapse probability of 0.1% was incorporated into the model based on sustained response reported by Bomback et al.^12^

^f^Cyclophosphamide effectiveness was calculated using a risk ratio from a meta-analysis by Xue et al comparing rituximab and cyclophosphamide.^15^

^g^No treatment-related serious adverse events were reported for Acthar® Gel in the reviewed studies; therefore, serious adverse events were not considered for Acthar® Gel.^2,8,12–14^

^h^All costs inflated to 2025 USD.

^i^Drug costs as of January 1, 2025.

Efficacy data for Acthar® Gel was derived from a phase Ib/II, open-label, dose-finding study, where patients were randomly assigned to receive 40 or 80 units subcutaneously for 3 to 6 months.^2^ Bomback et al reported sustained responses with Acthar® Gel.^12^ Considering the single study reporting a 1-year sustained response without relapse, and the absence of subsequent relapse reports, a conservative relapse probability of 0.1% was incorporated into the model. Rituximab effectiveness data originated from a prospective study of patients referred to a nephrology unit following prior treatment failures.^16^ Cyclophosphamide effectiveness was calculated using a risk ratio from a meta-analysis comparing rituximab and cyclophosphamide.^15^ Due to the lack of studies examining cyclophosphamide use in patients failing conventional therapies, this approach was utilized, acknowledging the similar efficacy and safety profiles of cyclophosphamide and rituximab. Specifically, we applied the risk ratio reported by Xue et al^15^ at 6 months (and at last follow-up) to the observed rituximab response probability via an odds-based transformation, using the following formula:

**Figure:**
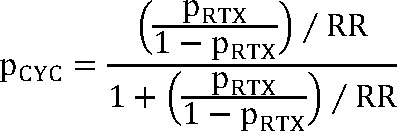


where p_CYC_ = probability of response with cyclophosphamide; p_RTX_ = probability of response with rituximab, and RR = risk ratio.

We further assumed that this odds-ratio conversion approximates true relative efficacy sufficiently for modeling purposes and that cyclophosphamide is now less commonly used than rituximab in this patient population. While cyclophosphamide has showed marginally higher efficacy, rituximab exhibits lower toxicity. Relapse probabilities for cyclophosphamide and rituximab were obtained from Xue et al.^15^

For cyclophosphamide^17^ and rituximab,^18^ the analysis only included serious adverse events (SAEs). Studies examining Acthar® Gel reported no treatment-related SAEs; consequently, SAEs were excluded from the Acthar® Gel evaluation.^2,8,12–14^

Pain in NS is multifaceted—encompassing nociceptive, neuropathic, and non-neuropathic elements—and is frequently observed in patients with iMN.^22^ In patients with ESRD, pain is widespread and contributes significantly to depression, disrupted sleep, reduced dialysis efficacy, and an increased tendency to withdraw from dialysis.^49^ Thus, the model incorporated pain prevalence using estimates from the literature.^21,22^ Similarly, depression is common in chronic kidney disease, including ESRD, and is linked to diminished quality of life and higher mortality^23^; accordingly, depression prevalence was also integrated into the model based on published data.^19,23^

Persistent proteinuria in NS due to iMN increases the risk of chronic complications, including hypertension, hyperlipidemia, and venous thrombosis.^50–52^ These conditions contribute to cardiac complications and accelerate the progression of chronic kidney disease.^50–52^ The probabilities of these complications were derived from published studies.^19,20^

In patients with iMN, kidney failure progression is primarily observed in relapsing and persistent NS health states. Given the limited data on Acthar® Gel and SoC, equivalent progression probabilities for both treatment groups were assumed.^3,24,25^ NS is also linked to a greater risk of mortality from complications, such as infections, kidney failure, and thrombosis, with 6-month excess mortality probabilities derived from the literature.^24,26^

**Cost inputs**: Wholesale acquisition costs (as of January 1, 2025), were sourced from the Micromedex Red Book (Merative).^27^ The cost of a 5 mL multidose vial of Acthar® Gel was $45 304; an average utilization of 7.86 vials per year (based on the specialty pharmacy dispensing data for 12 months ending December 31, 2023) was considered in the model. The total cost for SoC was calculated based on dosing information from the literature and clinical guidelines.^6,53^ Dosage was calculated considering an average body weight of 80.0 kg and a body surface area of 2.0 m². The annual cost of cyclophosphamide was $8554. Additionally, the rituximab cost calculation factored in the prices and market shares of the original biologic and its biosimilars, resulting in an annual cost of $103 242.

Administration costs of $4170 per year were applied exclusively to rituximab, given its intravenous delivery mode of administration.^54^ Both cyclophosphamide and rituximab require monitoring, with annual costs estimated at $480.^54^ Further, immunosuppressants require antibiotic prophylaxis, and trimethoprim/sulfamethoxazole use was considered, with an annual cost of $250.^27,55^ Additionally, anti-emetic costs were factored into the overall administration costs at an annual cost of $877.^56^

The model encompassed a variety of costs associated with the management of proteinuria in NS due to iMN across different health states. These costs included those for supplementary treatments (supportive care medications, immunosuppressants, plasmapheresis, and medications for pain medications, and antidepressants), iMN-related disease management (both inpatient and outpatient), complications, end-of-life care, and kidney failure management (dialysis or renal transplant) (**Table 1**). All cost estimates were derived from published literature and adjusted to 2025 US dollars using the US Consumer Price Index for Medical Care Services for Urban Consumers.^57^

Patients in every health state, except for those who had died, were assumed to receive supportive care medications to manage NS-related comorbidities such as deep vein thrombosis, hypertension, hyperlipidemia, and stroke. Utilization rates for these medications were as follows: angiotensin-converting enzyme inhibitors (ACEis) or angiotensin receptor blockers (ARBs) at 69.4%, beta-blockers at 22.2%, calcium channel blockers at 13.9%, diuretics at 73.6%, anticoagulants at 16.7%, and statins at 68.1%; derived from the Symphony Health database (January 1, 2016–December 31, 2022).

Patients in persistent NS health state were assumed to be treated with immunosuppressive therapies to control proteinuria. The treatment mix was distributed as follows: chronic systemic corticosteroids use at 25.0%,^19^ cyclosporine at 11.0%,^36^ tacrolimus at 11.1%^36^ and mycophenolate mofetil at 12.3%.^36^ Chronic corticosteroid use was presumed, meaning that all patients were at risk of experiencing corticosteroid-associated toxicity. Based on the published literature, SAEs for cyclosporine were distributed as 10.8% for cardiovascular events, 12.3% for infections, 3.1% for nephrotoxicity, and 1.5% for endocrine/metabolic issues.^18^ For tacrolimus, SAEs included 15.9% for reversible nephrotoxicity, 9.0% for irreversible nephrotoxicity, 43.1% for infections, and 22.7% for impaired fasting glucose/diabetes mellitus.^58^ Additionally, 3.0% of patients were assumed to undergo plasmapheresis.^59^ Data on the number of sessions^60^ and related expenses—covering acquisition, staffing, equipment, materials, and laboratory tests—were used to calculate the cost per session.^27–30,61^ The SAE profile for plasmapheresis was 4.9% for bleeding, 2.4% for catheter occlusion, 2.4% for deep vein thrombosis, and 2.4% for infections.^28^

Pain management strategies incorporated the use of nonsteroidal anti-inflammatory drugs (NSAIDs) and opioids.^31,32^ NSAID use was restricted to patients in the relapse phase because of concerns regarding worsening kidney function in persistent NS and renal failure health states.^62^ Utilization rates and toxicity risks for both NSAIDs and opioids were derived from the literature. In the relapse phase, 43.1% of patients used NSAIDs, while 52.8% used opioids [Symphony Health database (January 1, 2016–December 31, 2022)]. Opioids were estimated to be used by 20% of patients in the persistent NS health state,^63^ 40.6% of those on hemodialysis or peritoneal dialysis,^64^ and 60.0% of those with a kidney transplant.^65^ The likelihood of NSAID-related toxicity was 25%,^66^ and chronic opioid use was presumed to elevate the risk of opioid abuse. Costs associated with managing pain-medication toxicity were also obtained from published sources.^33,34^ Furthermore, costs for antidepressants were considered for patients who did not achieve a treatment response.^35^

Management costs of iMN encompassed inpatient and outpatient services for patients in persistent NS health state.^36^ Costs related to complications were assigned to patients with complications in persistent NS health state and disease progression,^39^ and end-of-life care costs for deceased patients were also considered.^40^ Management of ARF involved dialysis, with 89% of patients on hemodialysis and 11% on peritoneal dialysis.^67,68^ For ESRD, treatment options included dialysis (70%) or renal transplant (30%).^68^ Dialysis-related costs were applied to ARF patients, while ESRD patients incurred dialysis and/or transplantation costs.^37,38^ Additionally, costs associated with SAEs varied by treatment and were calculated based on the frequency and type of SAEs observed for each therapy, with SAE costs for SoC reported separately.^28,41–48^

### Cost-per-Response Analysis

The core analysis (base case) assessed one-year cost per response for Acthar® Gel and SoC treatments. This was computed by dividing the total healthcare costs per patient by the proportion of patients achieving resolved relapse within each treatment group. The cost per response was also evaluated at 2 and 3 years, as part of extended analyses. Additionally, the incremental cost-effectiveness was examined by calculating the difference in total healthcare costs per patient between the two treatment groups (incremental cost) divided by the difference in the proportion of patients achieving response between the two treatment groups (incremental response) across the specified time horizons.

Several key assumptions were made to reflect a population with refractory and persistent proteinuria in NS due to iMN and utilizing the best available data specific to this population. Acthar® Gel was modeled only in patients who have failed at least 2 prior therapies (including corticosteroids), consistent with its real-world clinical practice. In the absence of long-term relapse data for Acthar® Gel, an annual relapse rate of 0.1% was conservatively applied. Similarly, comparator therapies (off-label cyclophosphamide and rituximab) have been evaluated primarily in refractory cohorts with limited randomized evidence, only SAEs requiring hospitalizations were captured, consistent with FDA definitions and standard cost-effectiveness methodology and excluded less severe events. Further, SAEs were defined a priori as those necessitating hospitalization and therefore included only hospitalization-level events (eg, neutropenia, anemia) for cyclophosphamide and rituximab. Adverse events reported in Acthar® Gel studies (weight gain, hypertension, edema, fatigue, seizures) did not meet this hospitalization threshold, nor were they adjudicated as treatment-related, and thus were excluded. Deterministic and probabilistic sensitivity analyses were used to evaluate the baseline assumptions and influence of variations in model parameters to assess the robustness of the 1-year cost-per-response findings for Acthar® Gel vs SoC. A one-way deterministic sensitivity analysis was performed by altering each key model input by ±30% and drug acquisition costs by ±10%. A multivariable probabilistic sensitivity analysis was conducted using 5000 Monte Carlo simulations, where parameter values were randomly sampled from predefined probability distributions, allowing for a comprehensive evaluation of combined uncertainty. The selection of these distributions was informed by the inherent characteristics and limitations of the available data, ensuring that the full range of potential uncertainty was adequately captured.

## RESULTS

**Figure 2.**
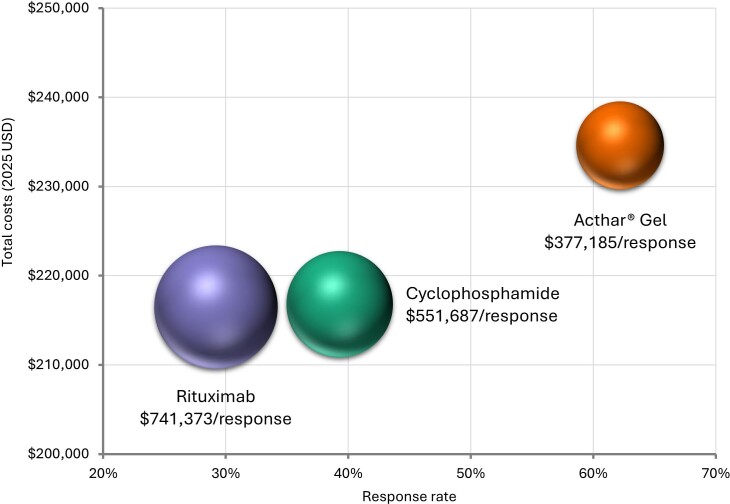
Cost per Response Over 1 Year From a US Payer Perspective Abbreviation: USD, US dollar. Note: Results are presented on a per-person basis. Size of the bubble in the plot represents cost-per-response.

**Figure 3.**
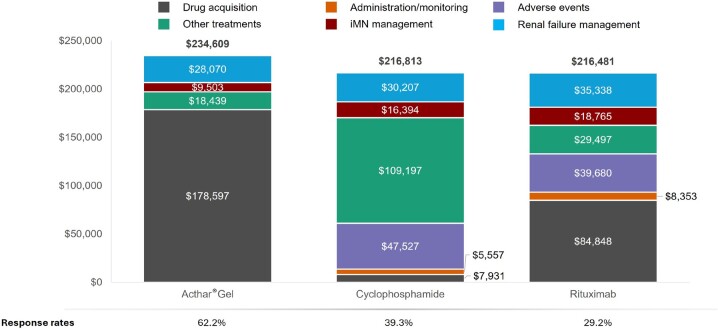
Overall Cost of Care and Response Rate Over 1 Year From a US Payer Perspective Abbreviation: iMN, idiopathic membranous nephropathy. Numbers in bold represent total costs. Results are presented on a per-person basis; All costs inflated to 2025 US dollars.

Over 1 year, Acthar® Gel exhibited a lower cost per response ($377 185) than cyclophosphamide ($551 687) and rituximab ($741 373) (**Figure 2**). Relative to cyclophosphamide, Acthar® Gel incurred an additional $17 796 per patient yet delivered a 22.9% higher response rate. Compared with rituximab, Acthar® Gel cost an additional $18 128 per patient while achieving a 33.0% higher response rate (**Figure 3**). Although Acthar® Gel’s drug acquisition cost was $170 666 higher than cyclophosphamide and $93 749 higher than rituximab, it resulted in lower overall medical costs resulting from other treatments, disease management, and renal failure and did not incur any treatment-related expenses (administration/monitoring and adverse events) over 1 year.

Other treatments include immunosuppressants (including monitoring and serious adverse events costs), supportive care treatments (antihypertensives, anticoagulants, and statins), plasmapheresis, pain medications, antidepressants; Disease management includes iMN-related inpatient and outpatient (physician office visits, emergency department, other outpatient); Renal failure management includes disease progression costs, including dialysis, renal transplant, and complications.

The cost-per-response benefit with Acthar® Gel over cyclophosphamide and rituximab was maintained at an extended time horizon of 2 and 3 years (**Table 2**). Specifically, Acthar® Gel had lower overall care costs and higher response rates relative to cyclophosphamide and rituximab at these extended time points. The patterns of overall care costs at extended time horizons mirrored those seen at 1 year (**Supplementary Table S1**).

**Table 2.** Cost per Response at 2 and 3 Years from a US Payer Perspective

**Timeframe and Intervention**	**Costs per Patient,^a^ $**	**Response Rate, %**	**Cost per Response,^b^ $**
2 years			
Acthar® Gel	298 671	60.7	492 044
Cyclophosphamide	378 855	39.6	956 705
Rituximab	381 399	29.6	1 288 510
3 years			
Acthar® Gel	366 775	59.2	619 552
Cyclophosphamide	540 223	36.3	1 488 218
Rituximab	550 043	27.3	2 014 810

^a^All costs inflated to 2025 US dollars.

^b^Results are presented on a per-person basis.

Acthar® Gel was a dominant treatment strategy in this economic evaluation vs unapproved treatments, cyclophosphamide and rituximab at 2 and 3 years, with lower costs and higher response rates (**Supplementary Table S2**).

**Figure 4.**
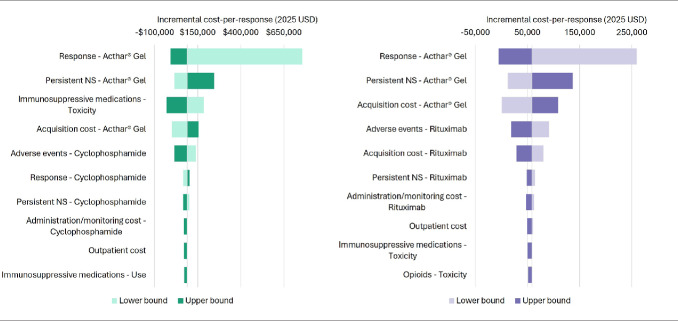
Key Drivers of Cost per Response Based on Deterministic Sensitivity Analysis of Acthar® Gel vs (**A**) Cyclophosphamide and (**B**) Rituximab Over 1 Year From a US Payer Perspective Abbreviations: NS, nephrotic syndrome; USD, US dollars. Notes: 1. Persistent NS health state refers to the state to which a patient transitions when a patient fails to show a significant clinical improvement following the full course of the prescribed initial treatment. 2. Bars represent the variation in the cost-per-response corresponding to high and low variation on a parameter’s point estimate. The “lower bound” represents the model outcome when a specific parameter is set to its minimum value within the specified range, while holding all other parameters at their base or central values. The “upper bound” represents the model outcome when a specific parameter is set to its maximum value within the specified range, while holding all other parameters at their base or central values. The vertical line dividing the lower and upper bound bars represents the base case cost-per-response when all inputs are set to their base case estimates. 3. Parameters are presented in a ranked order, with the most influential variables at the top. The length of the bars represents the magnitude of impact, and their direction (left or right) indicates whether the impact is positive or negative. If the impact of an input is positively related, then increasing the input estimate increases the cost-per-response (upper bound bar extends to the right; lower bound bar to the left) and vice versa. Conversely, if the impact of an input is negatively related, then increasing the input estimate decreases the cost-per-response (upper bound bar extends to the left; lower bound bar to the right) and vice versa.

The deterministic sensitivity analysis identified key contributors to cost per response over 1 year (**Figure 4**). The key contributors to cost per response for Acthar® Gel vs cyclophosphamide were the response rate and rate of persistent NS with Acthar® Gel, immunosuppressants-related toxicity, acquisition cost of Acthar® Gel, and cyclophosphamide-related SAEs. The key contributors to cost per response for Acthar® Gel vs rituximab were the response rate and rate of persistent NS with Acthar® Gel, the acquisition cost of Acthar® Gel and rituximab, and rituximab-related SAEs.

The probabilistic sensitivity analysis showed that Acthar® Gel was cost-effective in 77.4% of iterations vs cyclophosphamide and 91.3% of iterations vs rituximab at a willingness-to-pay threshold of $150 000 per response over 1 year (**Supplementary Figure S1**).

## DISCUSSION

Proteinuria plays a pivotal role in the progression of NS due to iMN and is a well-established risk factor for advancing renal disease.^3,7^ Addressing proteinuria in NS, particularly in cases refractory to conventional treatments, poses significant challenges due to the absence of uniformly accepted therapeutic approaches.^6–8^ This analysis assessed the economic value of treatment strategies for adults with proteinuria in NS due to iMN.

Over a period of 1 to 3 years, the findings indicated a more favorable cost per response for Acthar® Gel when compared with off-label therapies such as cyclophosphamide and rituximab. This economic advantage was consistently shown across a range of sensitivity analyses, reinforcing the robustness of the result. Notably, the analysis also showed that Acthar® Gel resulted in lower overall medical costs throughout the evaluation period. Consequently, Acthar® Gel presented as a dominant strategy, achieving both cost savings and improved response rates throughout the extended 2 and 3-year analysis timeframe.

Deterministic sensitivity analysis revealed that the higher response rate associated with Acthar® Gel was the primary factor driving its lower cost per response compared with SoC. Several other important cost drivers contributed to the more advantageous economic profile of Acthar® Gel relative to both SoCs. Other key drivers were a lower or similar probability of patients treated with Acthar® Gel progressing to the persistent NS health state, a higher likelihood of SAEs with SoC, and the higher initial acquisition cost of Acthar® Gel. Further analysis identified specific factors that were key drivers in the individual comparisons between Acthar® Gel and the off-label therapies. In the comparison with cyclophosphamide, a higher probability of immunosuppressant-related toxicity associated with cyclophosphamide was a significant factor. Similarly, the higher acquisition cost of rituximab was a key driver in its respective comparison with Acthar® Gel.

While the initial acquisition cost of Acthar® Gel was higher, the elimination of treatment-related costs associated with administration, monitoring, and the management of adverse events led to reduced overall healthcare spending. Further, the improved response rate translated to a decreased need for supportive and subsequent treatments, including immunosuppressive agents, plasmapheresis, and medications for pain and depression, disease management services, and costly interventions like dialysis and renal transplantation, effectively balancing the higher drug cost.

This study represents the first economic evaluation comparing the clinical outcomes and cost implications of treatment options for refractory proteinuria in NS due to iMN among patients who have failed on conventional therapies from a US payer perspective. It offers novel insights into a chronic, persistent, and refractory condition where evidence is currently limited. By mapping potential health states and transitions, the model provides a comprehensive framework for assessing alternative treatments’ short-term clinical and economic impacts on proteinuria in NS due to iMN. The transitions among health states in the model carry significant implications for both clinicians and payers. For example, the possibility of moving from a relapse to a response state highlights the differences in treatment effectiveness and underscores the need for personalized approaches. Similarly, the potential for cycling between relapse and response states emphasizes the chronic nature of proteinuria in NS due to iMN and the likelihood of ongoing treatment requirements. Transitions to acute or chronic renal failure further illustrate the potential severity of the disease and the consequent need for intensive interventions such as dialysis or transplantation, factors that incur substantial costs for payers. Moreover, the study’s findings provide clinicians and payers with important data that can be used to inform treatment decisions for adults who do not respond to conventional treatments. Given that proteinuria in NS due to iMN is challenging to manage, treatment selection involves an integrated approach, including the patient’s clinical profile, physician’s judgment, and the availability of resources. Finally, this analysis establishes a foundation for future research, including investigations into long-term clinical and economic outcomes and the identification of patient subgroups who may derive greater benefit from Acthar® Gel.

Optimal management of proteinuria in NS due to iMN aims to induce remission, mitigate complications, and delay renal failure, thereby lessening the economic impact. Consequently, it is crucial to evaluate therapies based on their capacity to deliver favorable clinical outcomes in a cost-effective manner.^69^ This analysis highlights the significant influence of Acthar® Gel’s greater response rate on its cost-per-response profile for the treatment of proteinuria in NS due to iMN. Despite a higher initial acquisition cost, the observed decrease in downstream healthcare costs underscores the potential for therapies with improved response rates to achieve treatment targets and generate cost efficiencies. This reinforces that interventions with better response rates can meet treatment objectives and provide cost savings for healthcare systems. The favorable cost per response of Acthar® Gel vs off-label SoC treatments could be further optimized through strategic pricing strategies (eg, rebates, discounts), further strengthening its value proposition as a potentially economically viable treatment option.

Immunosuppressant-related toxicity emerged as one of the major cost determinants in the comparison of Acthar® Gel and cyclophosphamide. Cyclophosphamide regimens, often also including systemic corticosteroids, carry a risk of long-term toxicity.^9,10^ SAEs associated with these SoC treatments also had a substantial impact on the economic analysis. Acthar® Gel showed an advantageous cost per response vs these off-label SoCs, signifying possible reductions in healthcare costs and improved clinical benefits through lower treatment-related toxicity rates.

This economic analysis acknowledges several inherent limitations. First, this analysis, conducted from a US payer perspective, may not be fully applicable to healthcare systems with alternative treatment protocols for adults with proteinuria in NS due to iMN. Second, the reliance on published observational studies for clinical and cost data may have introduced the possibility of deviations from real-world practice. Third, precisely quantifying the complex influence of proteinuria in NS due to iMN on health outcomes and resource consumption remains difficult due to limited data availability. Fourth, the model did not incorporate potential pricing adjustments from the manufacturer, such as discounts or rebates. Fifth, the absence of direct, randomized controlled trials and the off-label use of SoC treatments, supported by limited studies in refractory proteinuria in NS due to iMN, necessitated the use of indirect data, introducing a degree of uncertainty. Clinical efficacy inputs for Acthar® Gel are based on small Phase Ib/II trials and observational cohorts, rather than head-to-head randomized comparisons with off-label therapies, which may limit the precision of transition probability estimates. Although sensitivity analyses suggest that the assumed 0.1% relapse rate has minimal impact on cost results, it remains an area of uncertainty. A targeted, comprehensive literature search was conducted to capture data relevant to the specific population considered in this model; however, unpublished data or ongoing trials could alter the evidence base, and potential publication bias should be considered when interpreting study findings. Sixth, less severe AEs reported in some Acthar® Gel studies were not considered in this analysis as they did not meet the *a priori* definition of SAEs. While the selective inclusion of AEs could potentially lead to an underestimation of total costs, particularly for Acthar® Gel, it is essential to note that the exclusion of non-severe AEs also applies to the off-label comparator therapies. Furthermore, sensitivity analyses demonstrated that the inclusion of SAEs had a minimal impact on the overall cost-per-response outcomes, suggesting that this limitation likely has a limited effect on the study’s primary findings. Finally, this study was conducted from a US healthcare payer perspective, where decision-makers often focus on direct clinical outcomes. Furthermore, this analysis focused on short-term outcomes, primarily encompassing clinical benefits for a very specific population. Therefore, cost per response was a primary metric as response rates are the endpoints most directly measured and acted upon by payers in this setting. Further studies are needed to include cost per quality-adjusted life-years as an outcome, once long-term data and health utility data become available, allowing for direct comparability with other treatment economic evaluations.

Given the preliminary nature and reliance on modeled assumptions, the results are best viewed as hypothesis-generating. Further clinical studies would be valuable to help validate key inputs such as efficacy and relapse rates. Given the acknowledged limitations related to the limited randomized controlled trial data, relapse rates, heterogeneity in source populations, and SAE classification, the study conclusions rely on these assumptions and may change with the emergence of more robust clinical evidence. To address these limitations, the robustness of the results was evaluated through sensitivity analyses, which consistently validated Acthar® Gel’s advantageous cost-per-response profile. Head-to-head trials or registry data are needed to confirm the durability of response and capture the full safety profile.

The findings from this economic analysis should be interpreted in the context of the available data and underlying model assumptions and are intended to be viewed as preliminary and hypothesis-generating given the current evidence base in this population. Notwithstanding these limitations, the study suggests Acthar® Gel may represent a potentially value-based treatment strategy for adults with proteinuria in NS due to iMN. The observed favorable cost per response is primarily attributed to its enhanced response rate, which in turn minimizes the requirement for subsequent, high-cost interventions. This reduction in healthcare resource utilization effectively mitigates the initial drug expenditure, resulting in overall cost efficiencies. This analysis emphasizes the importance of a value-focused approach to treatment decisions for therapies that enhance patient outcomes and optimize healthcare resource allocation.

## CONCLUSIONS

This analysis indicates that, based on the current assumptions and clinical inputs, Acthar® Gel has the potential to be a cost-effective and value-based treatment strategy compared with unapproved SoCs for adults with refractory proteinuria in NS due to iMN, particularly for those who have failed multiple treatments. While the conclusions of this economic analysis are based on the available model inputs, they provide valuable preliminary insights and help generate hypotheses for further research, particularly given the limited data in this population. These results may inform clinical and payer decision-making in cases where standard therapies fail to achieve desired outcomes for a specific population. Additional research is needed to assess the long-term benefits of Acthar® Gel. Future research, including head-to-head trials or registry studies, is needed to confirm the durability of response and to fully characterize the safety profile.

## Supplementary Material

Online Supplementary Material
