# Supplementary material for: Cost per Response of Acthar® Gel vs Standard of Care for the Treatment of Proteinuria in Nephrotic Syndrome Due to Idiopathic Membranous Nephropathy Among Adults from the US Healthcare Perspective
Source: J Health Econ Outcomes Res. 2025 Aug 6;12(2):50–61. doi: 10.36469/001c.142078 (PMC12333935; doi:10.36469/001c.142078)
Supplement: Online Supplementary Material [file jheor_2025_12_2_142078_297142.pdf]

Journal of Health Economics and Outcomes Research

Online Supplementary Material

Cost per Response of Acthar® Gel vs Standard of Care for the Treatment of Proteinuria in Nephrotic Syndrome Due to Idiopathic Membranous Nephropathy Among Adults from the US Healthcare Perspective. *JHEOR*. 2025;12(2):50-61. doi:10.36469/jheor.2025.142078

Table S1: Cost Breakdown at 2 and 3 Years from a US Payer Perspective

Table S2: Incremental Cost per Response from a US Payer Perspective

Figure S1: Scatterplot Acthar® Gel vs Cyclophosphamide and Rituximab Over 1 Year from a US Payer Perspective

This supplementary material has been provided by the authors to give readers additional information about their work.

This is an open-access article distributed under the terms of the Creative Commons Attribution 4.0 International License (CCBY-4.0). View this license's legal deed at http://creativecommons.org/licenses/by/4.0 and legal code at http://creativecommons.org/licenses/by/4.0/legalcode for more information.

Table S1. Cost Breakdown at 2 and 3 Years from a US Payer Perspective

Cost Components[a,b]	Acthar Gel®, $	Cyclophosphamide, $	Rituximab, $
2 years			
Drug acquisition	172 803	11 578	116 173
Administration/monitoring	0	8 112	11 436
Adverse events	0	69 383	54 329
Other treatments[c]	34 490	172 139	59 005
Disease management[d]	17 244	29 111	33 916
Renal failure management[e]	74 134	88 532	106 540
3 years			
Drug acquisition	167 171	14 549	141 463
Administration/monitoring	0	10 193	13 926
Adverse events	0	87 185	66 156
Other treatments[c]	48 067	225 886	85 388
Disease management[d]	23 654	40 246	46 906
Renal failure management[e]	127 883	162 164	196 204

Abbreviation: iMN, idiopathic membranous nephropathy.
[a]Results are presented on a per-person basis
[b]All costs inflated to 2025 United States Dollars
[c]Other treatments include immunosuppressants (including monitoring and serious adverse events costs), supportive care treatments (antihypertensives, anticoagulants, and statins), plasmapheresis, pain medications, antidepressants
[d]Disease management includes iMN-related inpatient and outpatient (physician office visits, emergency department, other outpatient)
[e]Renal failure management includes disease progression costs, including dialysis, renal transplant, and complications

Table S2. Incremental Cost per Response from a US Payer Perspective

Time Frame and Intervention	Incremental Costs, $[a,b,c]	Incremental Response, %	Incremental Cost per Response, $[a,b]
1 year			
Acthar Gel® vs cyclophosphamide	17 796	22.9	77 712
Acthar Gel® vs rituximab	18 128	33.0	54 933
2 years			
Acthar Gel® vs cyclophosphamide	−80 184	21.1	Dominant
Acthar Gel® vs rituximab	−82 728	31.1	Dominant
3 years			
Acthar Gel® vs cyclophosphamide	−173 448	22.9	Dominant
Acthar Gel® vs rituximab	−183 268	31.9	Dominant

[a]All costs inflated to 2025 US dollars.
[b]Results are presented on a per-person basis.
[c]Negative incremental costs indicate that Acthar Gel® is cost-saving.

Figure S1. Scatterplot Acthar® Gel vs Cyclophosphamide and Rituximab Over 1 Year from a US Payer Perspective

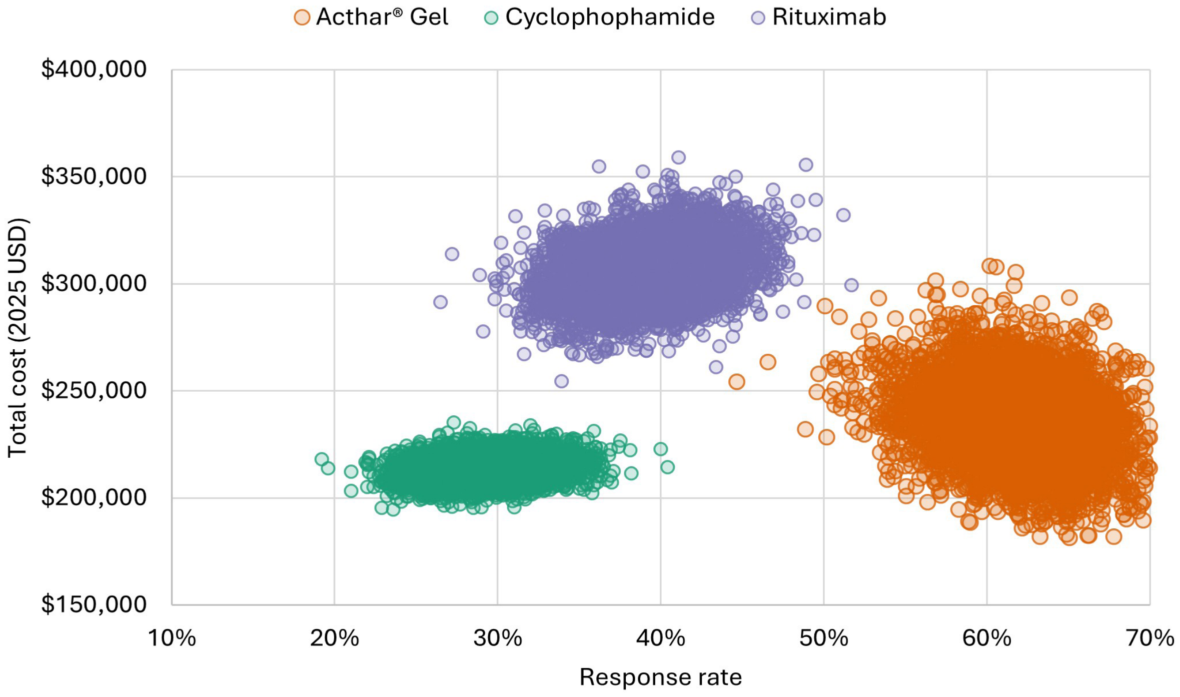

Abbreviations: USD, US dollars; WTP, willingness to pay.
Note: Willingness-to-pay threshold of $150 000 per response.
